# Genomes of Vibrio cholerae O1 Serotype Ogawa Associated with Current Cholera Activity in Pakistan

**DOI:** 10.1128/mra.00887-22

**Published:** 2022-11-30

**Authors:** Eby M. Sim, Elena Martinez, Grace A. Blackwell, David Pham, Gretschen Millan, Rikki M. A. Graham, Rajat Dhakal, Qinning Wang, Basel Suliman, Amy V. Jennison, Vitali Sintchenko

**Affiliations:** a Sydney Institute for Infectious Diseases, The University of Sydney, Sydney, New South Wales, Australia; b Centre for Infectious Diseases and Microbiology—Public Health, Westmead Hospital, Sydney, New South Wales, Australia; c Centre for Infectious Diseases and Microbiology Laboratory Services, Institute of Clinical Pathology and Medical Research, NSW Health Pathology, Sydney, New South Wales, Australia; d Department of Microbiology and Infectious Diseases, Liverpool Hospital, NSW Health Pathology, Sydney, New South Wales, Australia; e Q-PHIRE Genomics and Public Health Microbiology, Queensland Health Forensic and Scientific Services, Brisbane, Queensland, Australia; f School of Medical Sciences, Faculty of Medicine and Health, The University of Sydney, Sydney, New South Wales, Australia; University of Maryland School of Medicine

## Abstract

Here, this report presents two genomes of Vibrio cholerae O1 serotype Ogawa, recovered from cholera cases in Australia linked to travel to Pakistan in 2022. Their multidrug-resistant genotype represents the current activity of cholera within the seventh pandemic. One of the genome sequences was assembled using both short- and long-read sequences.

## ANNOUNCEMENT

Seven distinct pandemics associated with Vibrio cholerae O1 have been described since 1896 (1). The current seventh pandemic arose in 1961 with global spread via three separate waves and remains a public health burden for many countries experiencing localized outbreaks (2–5). There has been a recent upsurge of cholera cases in Pakistan amid the global COVID-19 pandemic (4, 6), and WHO recognized a risk of the outbreak spread associated with international travel (7). This report provides high-quality genome sequences recovered from human cases diagnosed with cholera linked to that region during this upsurge. Two isolates of V. cholerae O1 serotype Ogawa which were cultured from two unrelated cases of cholera in Australian residents in 2022 were subjected to whole-genome sequencing. Case 1 (Vch-N1252) was diagnosed in New South Wales (NSW) and had an indirect link to a recent traveler from Pakistan. Case 2 (Vch-Q4233) was diagnosed in Queensland (QLD) and reported recent travel to Pakistan. A nonresearch determination for activities of Vch-N1252 was granted by Health Protection New South Wales as it was performed under the NSW Public Health Act. An analysis of Vch-Q4233 was performed under the QLD Public Health Act, and analysis received Forensic and Scientific Services (FSS) Human Ethics Committee Clearance (HEC 22–26).

Both isolates were first identified as V. cholerae O1 biotype El Tor serotype Ogawa in NSW and QLD. Parameters from isolation through to whole-genome sequencing are listed in Table 1. Default parameters were used for all software unless otherwise specified. Short reads of both isolates were trimmed (SLIDINGWINDOW: 4:20, LEADING: 3, TRAILING: 3 MINLEN: 36 [Vch-Q4233] or 100 [Vch-N1252]) using Trimmomatic version 3.04 (8), and Vch-Q4233 was assembled using SPAdes version 3.12.0 with the inclusion of the “--careful” flag (9). Live base-calling and demultiplexing (“Trim barcodes” and “Mid read barcode filtering” enabled) were performed on a GridION device running MinKNOW version 21.10. Nanopore reads were filtered (--min_length 1000, --keep_percent 95) using Filtlong version 0.2.1 (https://github.com/rrwick/Filtlong). Hybrid assembly, along with the circularization and genomic rotation of Vch-N1252, was performed using Unicycler version 0.4.8 (10). Chromosome one was rotated to start with *dnaA*, while chromosome two was not rotated. Circularity was confirmed visually using Bandage version 0.9.0 (11). Genes conferring antimicrobial resistance (AMR) were detected using Abricate version 1.0.1 (https://github.com/tseemann/abricate) against AMRFinderPlus (12).

**TABLE 1:** Key parameters from isolation through to draft assembly of Vch-Q4233 and the hybrid assembly of Vch-N1252

Parameter	Information for:		
	Vch-Q4233	Vch-N1252	
		Short reads	Long reads
Isolation			
Specimen type	Isolate was referred via a pathology provider	Feces	
Initial detection		Allplex-GI^*a*^-bacteria (Seegene)	
Enrichment conditions		Alkaline peptone water at 37°C for 8 h	
Isolation conditions		Thiosulfate-citrate-bile salts-sucrose agar at 37°C for 48 h	
Isolate identification		MALDI-TOF MS^*b*^ (Bruker) and ID 32E (bioMérieux)	
Isolate maintenance	Thiosulfate-citrate-bile salts-sucrose agar at 37°C for 24 h		
DNA extraction			
Growth conditions	Horse blood agar at 37°C for 20 ± 2 h		
DNA extraction kt	QiaSymphony DSP DNA minikit (Qiagen)	DNeasy blood and tissue kit (Qiagen)	DNeasy UltraClean microbial kit (Qiagen)
Type of extraction	Spin column	Spin column	Spin column
Library prepn and sequencing			
Library prepn kit	Nextera XT (Illumina)	Nextera XT (Illumina)	Rapid barcoding kit (Oxford Nanopore Technologies)
Reagent kit/flow cell	Midoutput kit, 150 cycles	Midoutput kit, 150 cycles	R9.4.1
Sequencing platform	NextSeq 500	NextSeq 500	GridION
DNA input	N/A^*c*^	N/A	500 ng
Sequencing runtime	N/A	N/A	20 h 32 min
Sequencing and assembly statistics			
No. of reads^*d*^	2,439,076	3,559,816	111,064
Minimum read length (no. of bases)	36	100	1,000
Maximum read length (no. of bases)	151	151	36,440
Read length *N*_50_ (no. of bases)	150	150	5,310
Cumulative read length (no. of bases)	341,317,784	521,169,415	444,176,625
Sequencing depth (×)^*e*^	82.48	125.93	107.33
No. of contigs	176	N/A	2
Assembly length (no. of bases)	3,989,030	N/A	4,095,848
Assembly *N*_50_ (no. of bases)	77,137	N/A	3,046,907
Size of chromosome 1 (no. of bases)	N/A	N/A	3,046,907
Size of chromosome 2 (no. of bases)	N/A	N/A	1,048,941
G+C content (%)	47.61	N/A	Chr. 1, 48.39; Chr. 2, 47.88
Detected AMR genes	*dfrA1, sul2*, *strA*, *strB*, *floR*, *varG*, *catB9*	*dfrA1, sul2*, *strA*, *strB*, *floR*, *varG*, *catB9*	*dfrA1, sul2*, *strA*, *strB*, *floR*, *varG*, *catB9*
Accession no.			
Sequence Read Archive	SRR21074467	SRR21074466	SRR21074465
GenBank^*f*^	JANQBL000000000	N/A	CP102927, CP102928
NCBI RefSeq^*g*^	N/A	N/A	NZ_CP102927.1, NZ_CP102928.1

a GI, gastrointestinal.

b MALDI-TOF MS, matrix-assisted laser desorption ionization–time of flight mass spectrometry.

c N/A, not available.

d Counts of trimmed reads and filtered reads for short reads and long reads, respectively.

e Calculated using the assembly length of ASM836960v1.

f Assembly of Vch-Q4233 was annotated by the NCBI Prokaryotic Genome Annotation Pipeline version 6.3 [15].

g RefSeq sequences were annotated by the NCBI Prokaryotic Genome Annotation Pipeline version 6.2 [15, 16].

**FIG 1:**
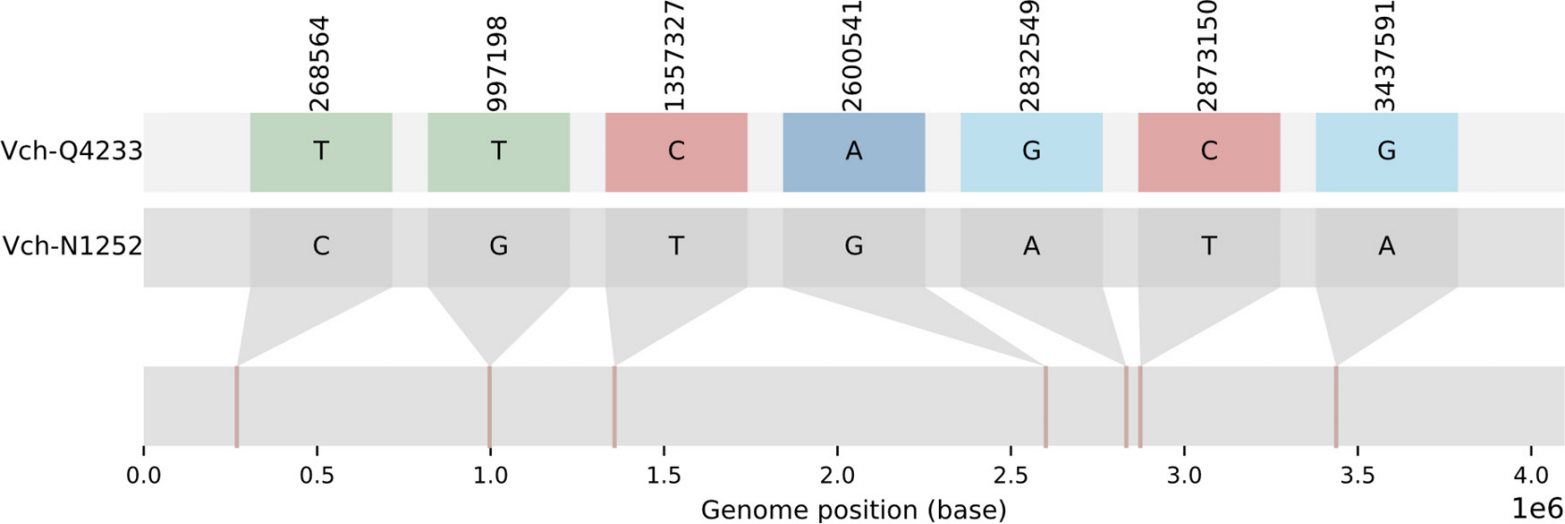
Genomic similarity between genomes of Vch-N1252 and Vch-Q4233. Graphical representation of a pair-wise alignment between Vch-N1252 and a pseudomolecule of Vch-N1252 with identified SNPs of Vch-Q4233 incorporated. The pseudomolecule was generated from an output from snippy version 4.6.0 (https://github.com/tseemann/snippy). The image was generated using snipit (https://github.com/aineniamh/snipit). Default settings were used for both programs.

Both genomes were related genetically with only seven single nucleotide polymorphisms (SNPs) separating them (Fig. 1), and both harbored the *ctxAB* (*ctxB7* allele) genes. The same set of AMR genes was detected in the assemblies of both isolates based on short reads, and the hybrid assembly of Vch-N1252 confirmed that *drfA1*, *sul2*, *strA*, *strB*, and *floR* were carried within an integrative and conjugative element, as reported previously (13). This submission is inspired by the call for a timely sharing of international genomic surveillance data (14) and may be helpful for monitoring the spread and control of the ongoing outbreak in Pakistan during the seventh pandemic of cholera and beyond.

### Data availability.

Reads and assemblies were deposited in SRA and GenBank, respectively. Accession numbers are provided in Table 1.
